# Hepatic wound repair

**DOI:** 10.1186/1755-1536-2-4

**Published:** 2009-09-25

**Authors:** Maurizio Parola, Massimo Pinzani

**Affiliations:** 1 Department of Experimental Medicine and Oncology and Interuniversity Center for Liver Pathophysiology, University of Torino, Torino, Italy; 2 Department of Internal Medicine and Center for Research, Transfer and High Education, University of Florence, Florence, Italy

## Abstract

**Background:**

Human chronic liver diseases (CLDs) with different aetiologies rely on chronic activation of wound healing that represents the driving force for fibrogenesis progression (throughout defined patterns of fibrosis) to the end stage of cirrhosis and liver failure.

**Issues:**

Fibrogenesis progression has a major worldwide clinical impact due to the high number of patients affected by CLDs, increasing mortality rate, incidence of hepatocellular carcinoma and shortage of organ donors for liver transplantation.

**Basic science advances:**

Liver fibrogenesis is sustained by a heterogeneous population of profibrogenic hepatic myofibroblasts (MFs), the majority being positive for α smooth muscle actin (αSMA), that may originate from hepatic stellate cells and portal fibroblasts following a process of activation or from bone marrow-derived cells recruited to damaged liver and, in a method still disputed, by a process of epithelial to mesenchymal transition (EMT) involving cholangiocytes and hepatocytes. Recent experimental and clinical data have identified, at tissue, cellular and molecular level major profibrogenic mechanisms: (a) chronic activation of the wound-healing reaction, (b) oxidative stress and related reactive intermediates, and (c) derangement of epithelial-mesenchymal interactions.

**Clinical care relevance:**

Liver fibrosis may regress following specific therapeutic interventions able to downstage or, at least, stabilise fibrosis. In cirrhotic patients, this would lead to a reduction of portal hypertension and of the consequent clinical complications and to an overall improvement of liver function, thus extending the complication-free patient survival time and reducing the need for liver transplantation.

**Conclusion:**

Emerging mechanisms and concepts related to liver fibrogenesis may significantly contribute to clinical management of patients affected by CLDs.

## Background

Human chronic liver diseases (CLDs) are characterised by reiteration of liver injury due to chronic infection by viral agents (mainly hepatitis B and C viruses) or to metabolic, toxin/drug-induced (alcohol being predominant) and autoimmune causes. This results in the chronic activation of the wound-healing response that represents the driving force for progressive accumulation of extracellular matrix (ECM) components, eventually leading to liver cirrhosis and hepatic failure. Accordingly, cirrhosis can be defined as an advanced stage of fibrosis involving the formation of regenerative nodules of parenchyma surrounded and separated by fibrotic septa, a scenario also characterised by significant changes in hepatic angioarchitecture [1-4].

Fibrosis progression is strictly related to the underlying cause of CLD and four distinct patterns of fibrosis progression (shown below) have been identified that are related to the 'topographic site' of tissue injury (as previously reported in detail [2]), the involvement of different populations of hepatic myofibroblast-like (MF) cells and the predominant profibrogenic mechanism [2,4,5].

### Bridging fibrosis

This is typically found in chronic viral hepatitis, considered as result of portal-central (vein) bridging necrosis, resulting primarily in the formation of portal-central fibrotic septa. Interface hepatitis, development of portal-portal septa and early changes in vascular connections with portal system complete this scenario.

### Perisinusoidal/pericellular fibrosis

This is typically found in non-alcoholic steatohepatitis (NASH) or associated with alcoholic aetiology (ALD); excess deposition of ECM is primarily in the spaces of Disse around sinusoids or groups of hepatocytes, leading to the two scenarios of 'capillarisation of sinusoids' or 'chicken-wire pattern', respectively.

### Biliary fibrosis

This is a pattern sustained by concomitant proliferation of reactive bile ductules and periductular MFs at the interface between portal areas and parenchyma, leading to the characteristic development of portal-portal fibrotic septa.

### Centrolobular fibrosis

This is secondary to conditions altering venous outflow, as in chronic heart failure, characterised by the development of central-central (vein) fibrotic septa.

### Clinical problems

Fibrogenesis progression has a major clinical impact because [2,4]: (1) millions of patients worldwide are affected by a form of CLD, and among these 25% to 30% are expected to develop significant fibrosis and cirrhosis; (2) liver cirrhosis is the most common non-neoplastic cause of death in Europe and USA among diseases interesting the gastrointestinal (GI) tract, and represents the seventh most common cause of death in western countries; (3) liver cirrhosis predisposes patients to hepatocellular carcinoma (HCC) that further increases mortality rate; (4) epidemiological analysis predict a peak for advanced CLD in the next decade, with an increased number of patients reaching end-stage disease and requiring liver transplantation.

### Relevant basic science context

Liver fibrogenesis is sustained by a heterogeneous population of profibrogenic hepatic MFs expressing a peculiar repertoire of antigens [5], including α smooth muscle actin (αSMA), which is expressed by the majority, but not all, of these cells. On the basis of tissue localisation and/or antigen profile different populations of MFs may be recognised: (a) activated, MF-like, hepatic stellate cells (HSC-MFs) found primarily in or around capillarised sinusoids of fibrotic/cirrhotic livers; (b) portal/septal MFs (PS/MFs) found in the connective tissue around expanded portal tracts or in the inner part of fibrotic septa; and (c) interface MFs (IF/MFs), found at the edge between fibrotic septa and the surrounding parenchyma.

Hepatic MFs can originate from different cellular sources: (1) activation of hepatic stellate cells [1-6] where HSC/MFs are likely to predominate in a pattern of 'perisinusoidal/pericellular fibrosis' and contribute to 'bridging fibrosis'; (2) activation of portal fibroblasts (that can give origin also to septal MFs), relevant in ischemic conditions and in obstructive cholestatic diseases and giving origin also to septal MFs; (3) MFs (IF/MFs and some portal MFs) may originate from recruitment of bone marrow derived cells [7] such as mesenchymal stem cells (MSCs) [8,9] and circulating fibrocytes [10]; and (4) MFs, with a significant number of cells reported to be αSMA negative, may also originate by means of epithelial to mesenchymal transition (EMT) of hepatocytes and/or cholangiocytes [11,12], although this possibility remains controversial.

**Figure 1:**
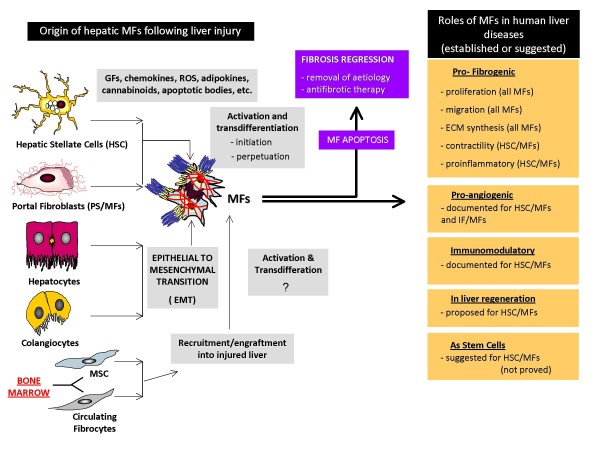
**Hepatic MFs may originate following a common process of activation/transdifferentiation or by EMT, from the indicated cellular sources**. Different roles, established or suggested, for hepatic MFs are also indicated.

Present knowledge on profibrogenic MFs is derived mainly from studies performed on human or rodent HSC/MFs [1-6,13,14]. Activation of HSCs in CLD progresses in sequential stages of initiation and perpetuation. Initiation is an early response stimulated by paracrine signals and leading to a transient and potentially reversible contractile and profibrogenic phenotype, characterised by rapid induction of platelet-derived growth factor (PDGF)β receptor and primed to respond to several growth factors and mediators that will be crucial in eliciting phenotypic responses operated by fully activated MF-like phenotype (perpetuation), including proliferation, migration/chemotaxis, contractility, excess deposition and altered remodelling of ECM (Figure 1).

### Experimental models or material: advantages and limitations

Major advances in our understanding of fibrogenic mechanisms come directly from experimental studies. Studies on primary cultures of rat and human HSCs have dissected mechanisms and signalling pathways controlling the process of activation and related major phenotypic responses, in a scenario [1-6] that identifies these cells as crucial cellular crossroads in a complex sinusoidal environment requiring tightly regulated autocrine and paracrine crosstalk, rapid responses to evolving ECM content, and selective responsiveness to the metabolic needs imposed by liver growth and repair. This also includes evidence suggesting that HSCs may be essential for hepatic progenitor cell amplification and differentiation and be involved in antigen presentation and induction of tolerance. Emerging evidence suggesting that hepatic MFs may also originate from portal fibroblasts, the EMT process and bone marrow-derived cells represent the major limitation to these studies that theoretically may not represent an absolute paradigm for all the fibrosis patterns.

Similarly, rodent models of CLD [1-6,13-22] including those taking advantage of knock-out or transgenic mice, have been instrumental in the mechanistic comprehension of major tissue, cellular and molecular mechanisms relevant to liver fibrogenesis as well as in identifying potential therapeutic targets. Although most of these pathogenic features have been confirmed in clinical conditions, major general limitations of animal models in relation to human CLDs [15,23] are represented by the fact that the most reliable rodent models of CLD usually do not completely match either the histopathology or selected pathophysiological features of the corresponding human condition. For example [23], most common rodent models of NASH lack the development of obesity and insulin resistance (methionine and choline deficient (MCD) diet) or significant progression towards fibrosis (high fat diet). Moreover, when these models (chronic CCl_4 _administration, bile duct ligation) are discontinued, an almost complete reversion even from cirrhosis is detected; a scenario that can hardly apply to human cirrhosis.

## Discussion of findings and relevant literature

Recent data in the literature from clinical conditions and experimental models [1-6,13-18] identify three major mechanisms able to elicit and sustain liver fibrogenesis, outlined below.

### Chronic activation of the wound-healing reaction

As in other fibrogenic disorders affecting different organs and systems, activation of the wound-healing reaction is the most common and relevant mechanism in hepatic fibrogenesis and is characterised by the following key features: (a) persistence of parenchymal damage with variable degree of necrosis and apoptosis; (b) presence of a heterogeneous inflammatory infiltrate including mononuclear cells and cells of the immune system; (c) activation of different types of ECM-producing and MF-like cells with marked proliferative, synthetic and contractile features; and (d) qualitative and quantitative changes of hepatic ECM, associated with limited or absent remodelling in the presence of a persistent attempt of hepatic regeneration [19].

Studies performed in the past two decades in animal models and human patients have outlined the role of several growth factors and cytokines (released by activated inflammatory cells, endothelial cells or by HSC and HSC/MFs) involved in the chronic wound-healing reaction and affecting the profibrogenic potential and phenotypic responses of HSC/MFs, including PDGF, transforming growth factor (TGF)β, connective tissue growth factor (CTGF), endothelin 1 (ET1), monocyte chemoattractant protein 1 (MCP1), and tumour necrosis factor (TNF)α [1-6,13-19]. Accordingly, several signalling pathways, transcription factors and related transcriptional gene regulation have been dissected and identified as involved in the process of activation of HSCs or in mediating phenotypic responses of MFs.

Major emerging concepts in this area are the following: (a) the role of adipokines and their possible involvement in non-alcoholic fatty liver disease (NAFLD) and NASH, for example, leptin, which is increased in obese patients with NASH and in other patients with CLDs, has been linked to fibrogenesis by experimental studies [20,21] and can affect profibrogenic behaviour of HSC/MFs [22,23]; (b) the role of hypoxia and angiogenesis that in CLDs have been suggested to favour fibrogenic progression [24], with HSC/MFs being able to both release proangiogenic cytokines and respond to them [24,25]; (c) liver fibrosis and, possibly, the initial stages of cirrhosis (unlikely for advanced cirrhosis) are potentially reversible in the presence of effective therapy and/or aetiology eradication [15,17], as regression of histopathology develops as a result of increased apoptosis of HSC/MFs and MFs and is paralleled by increased expression of interstitial collagenases by hepatic macrophages.

### Oxidative stress and related reactive intermediates

Oxidative stress has been detected in all major clinical conditions of CLDs and rodent experimental models, representing the predominant profibrogenic mechanism in NAFLD/NASH and ALD [18,23]. Increased generation of reactive oxygen species (ROS) and other reactive intermediates and decreased efficiency of antioxidant defences actively contributes to parenchymal cell death and to excess tissue remodelling and fibrogenesis [1-6,14,15,18,23]. ROS and other reactive mediators such as 4-hydroxynonenal (HNE) can be generated outside MFs being released either by activated inflammatory cells or hepatocytes damaged by the specific aetiological agent or condition. ROS and HNE can affect the profibrogenic response of MFs by modulating proliferation, synthesis of ECM, migration and chemotaxis, as well as ECM remodelling. Moreover, ROS generation within HSC/MFs has been reported to occur in response to profibrogenics including PDGF, angiotensin II and the adipokine leptin [26], being equally able to positively modulate profibrogenic signalling pathways through involvement of nicotinamide adenine dinucleotide phosphate (NADPH) oxidase [18,26], including ROS generation following phagocytosis of apoptotic bodies from damaged hepatocytes by HSC/MFs [27].

A final concept to mention is that the immune response triggered by oxidative stress may have a significant role in the progression of ALD, in the worsening of chronic hepatitis C by alcohol intake and, possibly, even in the progression of NAFLD [28].

### Derangement of epithelial-mesenchymal interactions and epithelial-mesenchymal transition in cholangiopathies

Cholangiopathies in adult and paediatric patients are characterised by cholestasis, necrotic or apoptotic loss of cholangiocytes, cholangiocyte proliferation (that is, ductular reaction) as well as portal/periportal inflammation and fibrosis. Intense proliferation of cholangiocytes is associated with recruitment of portal fibroblasts and HSCs followed by parenchyma invasion and biliary fibrosis. This scenario is dominated by an intense crosstalk between mesenchymal cells and cholangiocytes, the latter able to secrete chemokines (interleukin (IL)6, TNFα, IL8, MCP1) and profibrogenic factors (PDGF-BB, ET1, CTGF, TGFβ2) [2], and, as recently proposed, by a process of 'epithelial to mesenchymal transition' involving cholangiocytes and possibly driven by TGFβ [3,4,12].

## Take-home messages

### Basic science advances

Liver fibrogenesis is sustained by a heterogeneous population of profibrogenic hepatic MFs, the majority being positive for αSMA that may originate from different cellular sources including hepatic stellate cells and portal fibroblasts following a process of activation, bone marrow derived cells recruited to damaged liver and, in a still controversial method, by a process of EMT involving cholangiocytes and hepatocytes.

Present knowledge on hepatic MFs is derived mainly from studies performed on activation of HSC in CLDs, that progress in sequential stages of initiation and perpetuation. Initiation is an early response stimulated by paracrine signals leading to a transient, potentially reversible, contractile and profibrogenic phenotype, characterised by rapid induction of PDGFβ receptor and primed to respond to growth factors and mediators crucial in eliciting phenotypic responses operated by fully activated MF-like phenotypes (perpetuation), including proliferation, migration/chemotaxis, contractility, excess deposition and altered remodelling of the ECM.

Recent data in the literature from clinical conditions and experimental models have identified, at the tissue, cellular and molecular level, three major mechanisms able to elicit and sustain liver fibrogenesis: (a) chronic activation of the wound-healing reaction, (b) oxidative stress and related reactive intermediates, and (c) derangement of epithelial-mesenchymal interactions.

### Clinical science advances

The elucidation of the cellular and molecular mechanisms regulating fibrogenesis in chronic liver disease have provided relevant clinical advances in the identification of diagnostic and therapeutic strategies.

Several biomarkers, related to the biology of fibrogenesis are currently validated for their use as diagnostic and prognostic indicators of disease progression in different chronic liver diseases. Some of these markers are likely to replace or integrate the use of repeated liver biopsies in the follow-up of patients undergoing treatment. In addition, several potentially effective antifibrotic compounds have been identified, as previously detailed [2], and are awaiting clinical testing in large clinical trials.

### Relevance to clinical care

In the past decade, practicing hepatologists have finally directed their attention to hepatic fibrosis and its clinical implications, including the possibility of regression obtained by specific therapeutic interventions. Therefore, the issue of 'fibrosis regression' represents a current hot topic.

Since the degree of fibrosis in a non-cirrhotic liver is devoid of corresponding clinical manifestations, the main endpoint should be downstaging or, at least, stabilising fibrosis (that is, inducing a lack of progression). In other words, adequate therapy might ensure that the patient will die of other causes 'with' liver fibrosis rather than dying 'of' cirrhosis.

In cirrhotic patients, the reduction of fibrosis within cirrhotic liver tissue would lead to a reduction of portal hypertension and of the consequent clinical complications and to an overall improvement of liver function, thus extending the complication-free patient survival time and reducing the need for liver transplantation.

### Caution, critical remarks and recommendations

Although the relevant advances in basic knowledge on fibrosis obtained in the last 20 years have greatly promoted clinical applications pivotal for patient management, caution should be applied when translating basic concepts into clinical practice. Indeed, the search for effective antifibrogenic strategies is based on the knowledge gained in *in vitro *experiments and in experiments employing animal models of liver fibrosis. Besides the obvious consideration that *in vitro *data do not fully capture the situation in the whole organ and organism, it is also clear that none of the available animal model faithfully reflects human liver disease. Therefore, definite conclusions can be drawn only by clinical trials performed in humans.

In addition, while it is clear that the introduction of antifibrotic agents would represent a great therapeutic advantage, it is also absolutely clear that the best antifibrogenic treatment would be any strategy able to eliminate the primary cause of parenchymal damage, metabolic overload or excessive oxidative stress. Since the fibrogenic process is in essence a compensatory phenomenon aimed at maintaining a sufficient tissue continuity and cohesion in the presence of a continuous microscopic parenchymal collapse, it would be erroneous to attempt to cure fibrogenic CLDs only with antifibrogenic drugs.

## Future developments

A large effort is currently directed at identifying gene polymorphisms conditioning the rate of fibrosis progression in CLDs. The applications of 'omics' platforms (genomics, proteomics, metabolomics, and so on) has already generated an enormous amount of data that now needs to be analysed, framed in the clinical features of the disease and validated in larger cohorts. It is still unclear how these findings will translate to application with clinical utility in everyday practice. Regardless, molecular markers of the progression of fibrosis could help define new end points during antiviral therapy. Therefore, gene changes could be new markers of the progression of fibrosis during antiviral treatment. In addition, many of the upregulated genes identified in gene expression studies would represent potential molecular targets for the development of antifibrotic drugs.

## Abbreviations

ALD: alcoholic liver disease; CCl_4 _: carbon tetrachloride; CLDs: chronic liver diseases; CTGF: connective tissue growth factor; ECM: extracellular matrix; EMT: epithelial to mesenchymal transition; ET1: endothelin 1; GI: gastrointestinal; HCC: hepatocellular carcinoma; HNE: 4-hydroxynonenal; HSCs: hepatic stellate cells; HSC/MFs: activated, myofibroblast-like HSCs; IF/MFs: interface myofibroblasts; IL: interleukin; MCD: methionine and choline deficient; MCP1: monocyte chemoattractant protein 1; MFs: myofibroblasts; MSC: mesenchymal stem cells; NAFLD: non-alcoholic fatty liver disease; NASH: non-alcoholic steatohepatitis; PDGF: platelet-derived growth factor; PS/MFs: portal-septal myofibroblasts; ROS: reactive oxygen species; SMA: smooth muscle actin; TGFβ: transforming growth factor β; TNFα: tumour necrosis factor α.

## Competing interests

The authors declare that they have no competing interests.

## Authors' contributions

The two authors contributed equally to this review.
